# Baseball as a Safety Valve

**Published:** 1907-12

**Authors:** 


### BASEBALL AS A SAFETY VALVE.

---

The fundamental reason for the popularity of baseball is the fact that it is a national safety-valve. Voltaire says that there are no real pleasures without real needs. Now a young, ambitious and growing nation needs to "let off steam." Baseball furnishes the opportunity. Therefore it is a real pleasure.

But the outsider comprehends nothing of this. "Baseball," he argues loftily, "is a game for people whose minds are vacant, whose imagination is dull, who, of necessity, seek diversion because they have not enough soul leavening to be company for themselves."

In the face of what occurred at the opening game at the Polo grounds this year, the enthusiast hardly knows how to gainsay this aspersion. Commissioner Bingham having unexpectedly withdrawn all police protection, a whole army of fanatics—estimated at 15,000—charged on the field just when New York was on the point of overhauling Philadelphia. What did that throng care for victory or defeat! Who was John McGraw pleading that he might finish the game, when 15,000 mortal dynamos surcharged with pent-up emotion, energy and democratic enthusiasm were bent upon expressing themselves! This way and that swept the multitude—fans, bugs and rooters—pommeling one another with cushions, jubilating, yelling, making a seive of the welkin—physically and mentally getting everything "off the system." That is what baseball does for humanity. It serves the same purpose as a revolution in Central America, or a thunder storm on a hot day.—Allen Sangree, in September Everybody's.

